# Co-Stimulation of BCR and Toll-Like Receptor 7 Increases Somatic Hypermutation, Memory B Cell Formation, and Secondary Antibody Response to Protein Antigen

**DOI:** 10.3389/fimmu.2017.01833

**Published:** 2017-12-19

**Authors:** Diana P. Castiblanco, Robert W. Maul, Lisa M. Russell Knode, Patricia J. Gearhart

**Affiliations:** ^1^Laboratory of Molecular Biology and Immunology, National Institute on Aging, National Institutes of Health, Baltimore, MD, United States

**Keywords:** memory B cell, BCR, toll-like receptor 7, germinal center, somatic hypermutation, vaccine

## Abstract

The goal of immunization is to produce both a flood of antibodies to neutralize antigen and memory cells to accelerate the secondary response. To enhance the generation of memory B cells, we examined the effect of co-engaging BCR and toll-like receptor (TLR) 7 receptors by immunizing mice with a hapten-protein antigen, NP-CGG, and a ligand, R837 (imiquimod). During the early and late primary responses, there was no augmentation with R837 on the number of germinal center B cells or serum antibody. However, in the niche of germinal centers, R837 increased somatic hypermutation in the canonical V_H_1-72 gene that encodes NP-specific antibody. Increased mutation was not due to enhanced expression of activation-induced deaminase, but was likely a result of selection for high-affinity B cells with altered codons in the gene. This correlated with the appearance of antigen-specific B cells with a memory phenotype, which was intrinsic to TLR7 on B cells. To determine if these memory cells produced a recall response after a secondary challenge, spleen cells from mice that were immunized with NP-CGG and R837 were adoptively transferred into muMT recipients, and boosted with NP-CGG. Cells from mice that initially received both antigen and R837 generated a robust increase in germinal center B cells, plasmablasts, plasma cells, and serum antibody, compared with their cohorts who received antigen alone. These results support the use of co-immunization with TLR7 ligands to promote vigorous memory B cell responses to protein antigens.

## Introduction

Upon infection, the humoral response initiates a germinal center reaction which produces high-affinity antibodies and memory B cells. While antibodies play a crucial role in immediate clearance of pathogens, memory B cells protect upon re-encounter with pathogens. B cells can be activated through both the immunoglobulin receptor (BCR) and toll-like receptors (TLRs). TLRs, which are expressed in B cells, dendritic cells, macrophages, and neutrophils have been shown to affect antibody secretion and memory formation (1, 2). Some specific TLRs are TLR4 which responds to lipopolysaccharide (LPS), TLR7 which recognizes single-strand RNA, and TLR9 which binds CpG DNA. All three signal through the MYD88 adaptor protein, which is upregulated in germinal center B cells after TLR signaling (3). Stimulation through MYD88, TLR7, or TLR9 promotes autoimmune diseases, presumably by binding to RNA (TLR7), or DNA (TLR9) released from apoptotic cells (4–9). However, there is controversy over whether activation through TLR4 enhances antibody responses. In MYD88-deficient mice, an early report (10) showed that there was less antibody produced during a primary immunization with protein antigen and LPS, whereas later reports (3, 11, 12) indicated that antibody levels in MYD88-deficient mice were relatively normal after B cells were concomitantly stimulated with antigen and TLR4 ligands. Thus, it is not clear if immunization protocols using TLR4 agonists as adjuvants are beneficial.

In contrast, immunization with viruses which engage both BCR and TLR7 has been shown to magnify the humoral immune response. Studies utilizing mouse viruses, like Friend and lymphocytic choriomeningitis, reported that TLR7 signaling in B cells was necessary for viral clearance (13–15). Immunization with virus generated a robust antibody response and germinal center formation, which was absent in *Tlr7^−/−^* mice. While these studies indicated the importance of co-engagement of BCR and TLR7, the complex nature of viral antigens containing both protein and single-strand RNA complicated interpretations of how the increase was generated. Interestingly, co-stimulation of both TLR4 and TLR7 generated synergistic increases in antibody and memory B cells, compared with activation through either TLR alone (16). However, the question of whether a simple TLR7 agonist can amplify the response to protein antigens is poorly understood, particularly at the memory cell level. To address this conundrum, we used a reductionist approach of immunizing mice with a well-defined protein antigen (4-hydroxy-3-nitrophenyl)acetyl-chicken gamma globulin (NP-CGG) (17, 18), in the absence or presence of R837, also known as imiquimod. Imiquimod, an imidazaquinoline amine analog similar to guanosine, has anti-viral activity by activating immune cells *via* TLR7 (19). We observed no effect of BCR and TLR7 co-stimulation during the early and late primary responses in terms of frequency of germinal center B cells and antibody secretion. However, within germinal centers, R837 coordinated an increase in somatic hypermutation and cells with high-affinity mutations, which correlated with an expanded production of memory cells. During antigen recall, these memory B cells were surprisingly robust in magnifying the response, leading to increased germinal center cells, plasmablasts, plasma cells, and serum antibody after antigen challenge.

## Materials and Methods

### Mice

C57BL/6J, muMT (B6.129S2-Ighmtm1Cgn/J), and *Tlr7^−/−^* (B6.129S1Tlr7tm1Flv/J) mice were purchased from Jackson Laboratories, and bred in the National Institute on Aging animal colony. Mice of both sexes were used at 2–3 months of age. Mice were immunized intraperitoneally with 100 µg of 4-hydroxy-3-nitrophenyl)acetyl conjugated to chicken gamma globulin (NP_30_-CGG, BioSearch Technologies), which was emulsified in alum (Thermo Fisher Scientific), and with 30 µg of R837 (Sigma-Aldrich) in phosphate-buffered saline. Secondary boosts contained 50 µg of NP-CGG in alum. Animal protocols were reviewed and approved by the Animal Care and Use Committee at the NIA.

### Flow Cytometry

Single cell suspensions were prepared from spleens and stained with fluorochrome-conjugated antibodies. Germinal center B cells (B220^+^GL7^+^) were stained with FITC-labeled anti-B220 (RA3-6B2; Thermo Fisher Scientific) and Alexa Fluor 647-labeled anti-GL7 (GL7; BioLegend). Plasmablasts (B220^+^CD138^+^) and plasma cells (B220^−^CD138^+^) were stained with FITC anti-B220 and APC-labeled anti-CD138 (281-2; BioLegend). Mutated memory cells (NIP^+^B220^+^CD80^+^CD35^lo^) were visualized with FITC-labeled NIP_15_-bovine serum albumin (BSA) (Biosearch Technologies), PerCP-labeled B220 (RA3-6B2; Biolegend), PE-labeled CD80 (16-10A1; BD Biosciences), and BV 421-labeled anti-CD21/35 (7E9; Biolegend).

### ELISA

Microtiter plates were coated with NP_30_-BSA or NP_2_-human serum albumin (HSA) (Biosearch Technologies). Serum from unimmunized, day 14, or day 40 immunized mice was serially diluted fourfold, and used at a dilution of 1:25,600. Anti-NP antibodies were detected with goat anti-mouse IgG1 as described (20). Serum from muMT recipients after adoptive transfer was diluted 1:8 and tested against NP_30_-BSA. Higher dilutions did not give a signal above background, and the limited amount of antibody precluded analysis of binding to NP_2_-HSA.

### V_H_1-72 Mutations and AID Transcripts

RNA was extracted from day 14 germinal center B cells, and cDNA was synthesized using SuperScript III Reverse Transcriptase (Thermo Fisher Scientific). The rearranged V_H_1-72 gene joined to Cγ1 was amplified using Taq polymerase (Takara Bio USA) with the following nested primers: first set (leader), V_H_1-72 forward 5′ CATGCTCTTCTTGGCAGCAACAGC and Cγ1 (first exon) reverse 5′ GTGCACACCGCTGGACAGGGATCC, and second set: (framework region 1) V_H_1-72 forward 5′ CAGGTCCAACTGCAGCAG and Cγ1 (first exon) reverse 5′ AGTTTGGGCAGCAGA. The PCR products were cloned and sequenced; only sequences with unique CDR3 joins and mutations were counted. AID was measured by qPCR as described (21).

### Adoptive Transfers

For antigen-specific mutated memory cells arising during the primary response, naïve splenic B cells from C57BL/6 or *Tlr7^−/−^* mice were collected by negative selection with anti-CD43 and anti-CD11b magnetic beads (Miltenyi Biotec). 15–30 × 10^6^ B cells in 100 µl were injected into muMT recipients *via* tail-vein injection, and mice were immunized 1 day later with NP-CGG or NP-CGG plus R837. Recipient spleens were harvested 14 days later and gated on B220^+^NIP^+^ cells; the CD80^+^CD35^lo^ population of antigen-specific cells was then analyzed. For memory recall transfer of spleen cells, C57BL/6 mice were immunized with NP-CGG in the absence or presence of R837. Forty days later, 15–30 × 10^6^ total spleen cells from the immunized mice in 100 µl were injected into muMT mice *via* tail-vein injection. One day after transfer, muMT recipients were boosted with NP-CGG. Five days following immunization, muMT mice were sacrificed, and spleens and sera were analyzed.

## Results

### TLR7 Signaling Did Not Increase Germinal Center B Cells or Antibody Secretion during Early and Late Primary Responses to NP-CGG

**Figure 1:**
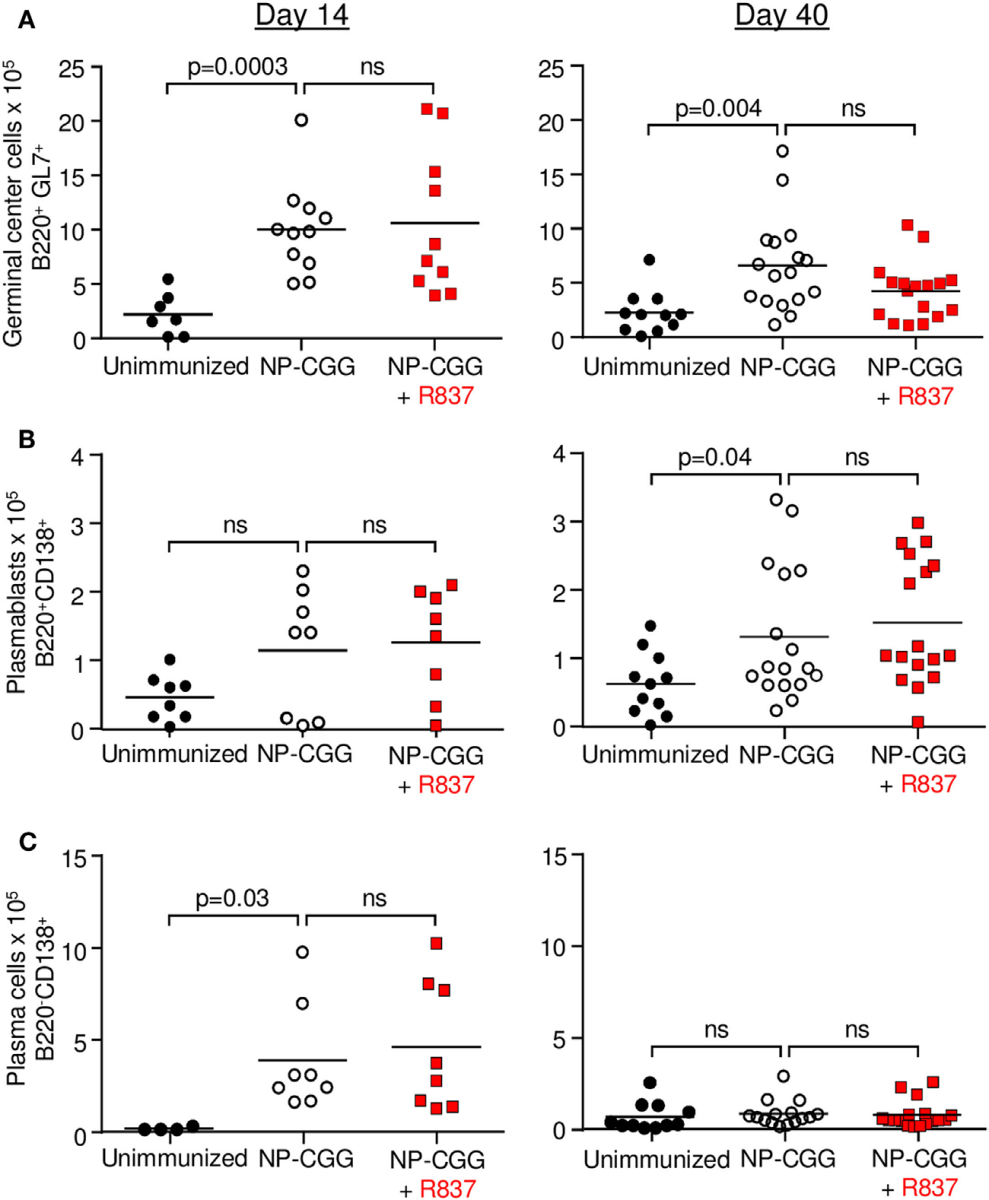
Toll-like receptor 7 signaling did not affect the frequency of splenic B cells during the early (day 14) and late (day 40) primary responses to NP-CGG as measured by flow cytometry. Mice were either unimmunized (black circles), immunized with NP-CGG (white circles), or immunized with NP-CGG plus R837 (red squares). **(A)** Germinal center B cells. **(B)** Plasmablasts. **(C)** Plasma cells. Each symbol represents the value from a single mouse, with four independent experiments of one to five mice each. Bar indicates mean; *p*-value determined by two-tailed Student’s *t*-test; ns, non-significant.

To investigate if co-stimulation of BCR and TLR7 enhanced B cell responses, mice were analyzed at several time points after immunization. The data in Figure 1 summarize results from days 14 to 40, which measured the effect during the early and late phases of a primary immunization. Analyses were also made at days 5 and 28, and showed no difference when R837 was included (data not shown). Similarly, long-lived plasma cells in bone marrow were analyzed by ELISPOT on days 5, 14, 28, and 40, and there was no difference when R837 was included (data not shown). After immunization, antigen-activated B cells enter germinal centers to divide, undergo mutation, and selection, and then become plasmablasts and plasma cells which secrete antibody. For germinal center B cells (B220^+^GL7^+^), immunization with NP-CGG alone produced a robust response on day 14 as quantified by flow cytometry, and by day 40, the number of cells decreased by about 50% (Figure 1A). However, there was no significant difference on either day when R837 was included in the immunization. For splenic plasmablasts (B220^+^CD138^+^), immunization did not generate an increase in cell numbers by day 14. By day 40, the numbers were modestly increased but were not augmented by R837 (Figure 1B). For splenic plasma cells (B220*^−^*CD138^+^), immunization with NP-CGG alone produced an increase on day 14, which was not enhanced by R837. The plasma cell count then waned by day 40 for both immunizations (Figure 1C).

**Figure 2:**
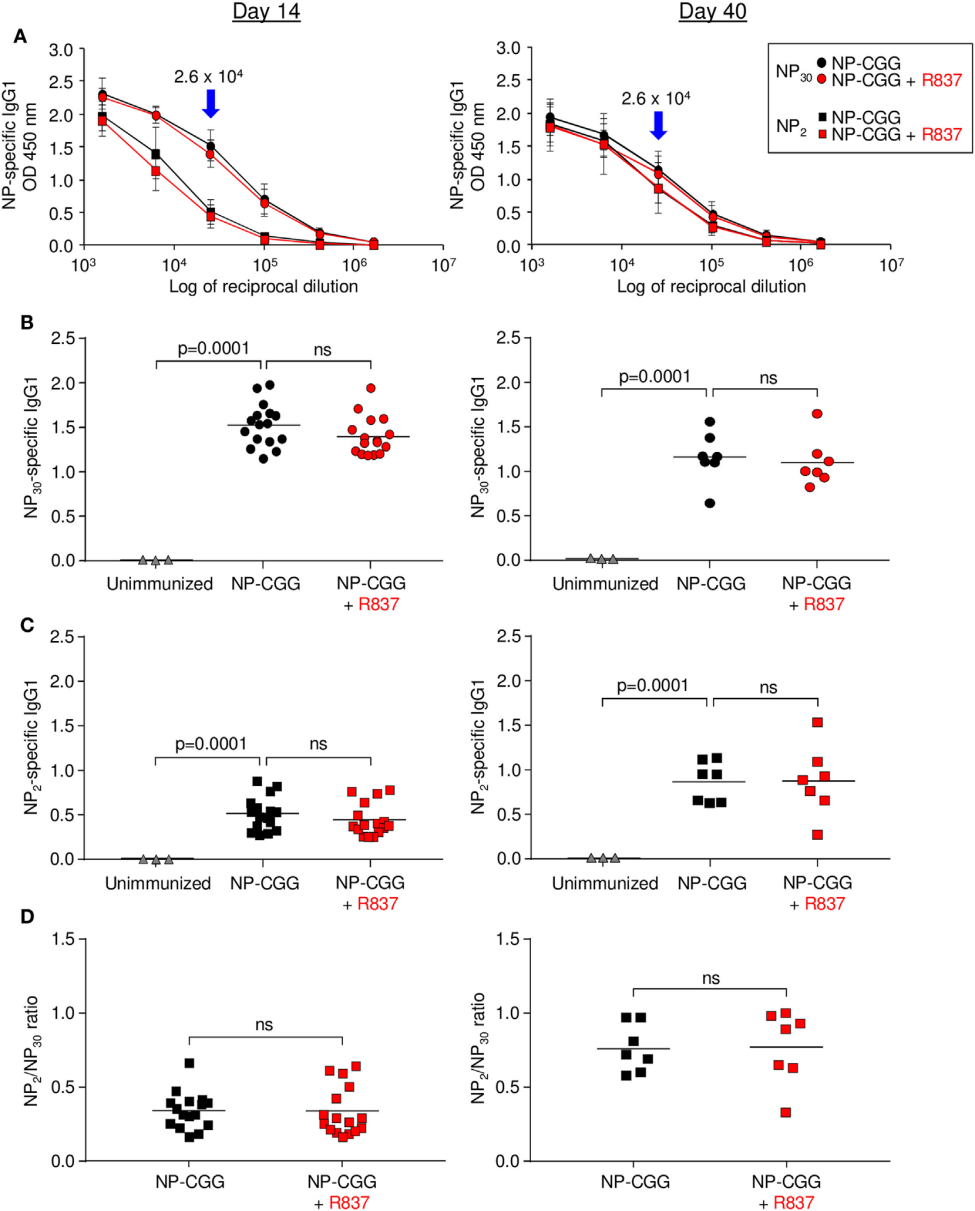
Effect of R837 on serum NP-specific IgG1 as measured by ELISA on days 14 and 40. **(A)** Serial dilutions of antibody binding to NP_30_-bovine serum albumin (BSA) or NP_2_-human serum albumin. Blue arrows mark the chosen dilution used for subsequent experiments. Circles and squares represent the average of individual samples with three to six independent experiments of two to three mice each. Error bars represent SD. **(B)** High- and low-affinity antibody binding to NP_30_. Each symbol represents serum from a single mouse. Triangles, sera from unimmunized mice. Bar indicates mean; *p*-value measured by two-tailed Student’s *t*-test; ns, non-significant. **(C)** High-affinity antibody binding to NP_2_. **(D)** Ratio of NP_2_/NP_30_ values.

For antibodies, NP-specific IgG1 was measured by ELISA in serum taken on days 14 and 40. As shown in Figure 2A, serum dilutions were performed after immunization with NP-CGG or NP-CGG plus R837. A 1:25,600 dilution was chosen for antibody concentrations, which was in the linear range of binding to NP_30_-BSA, which detects high- and low-affinity antibodies, or NP_2_-HSA, which binds to high-affinity antibodies. Immunization with NP-CGG produced robust increases in IgG1 antibody at both time points, but there was no additional effect with R837 for antibodies binding to NP_30_-BSA (Figure 2B) or NP_2_-HSA (Figure 2C). However, regardless of TLR7 signaling, there was an overall increase in high-affinity antibodies by day 40 compared with day 14 as measured by the ratio of NP_2_/NP_30_ binding, which has been correlated with affinity maturation (22). A comparison of ratios with both immunization protocols showed an increase on day 40 compared to day 14, suggesting affinity maturation with time (Figures 2A,D). Similar results were seen when IgG2c, an isotype that requires MYD88 signaling (12, 23), was measured (data not shown).

### Co-Stimulation Increased Somatic Hypermutation in Germinal Center B Cells

**Figure 3:**
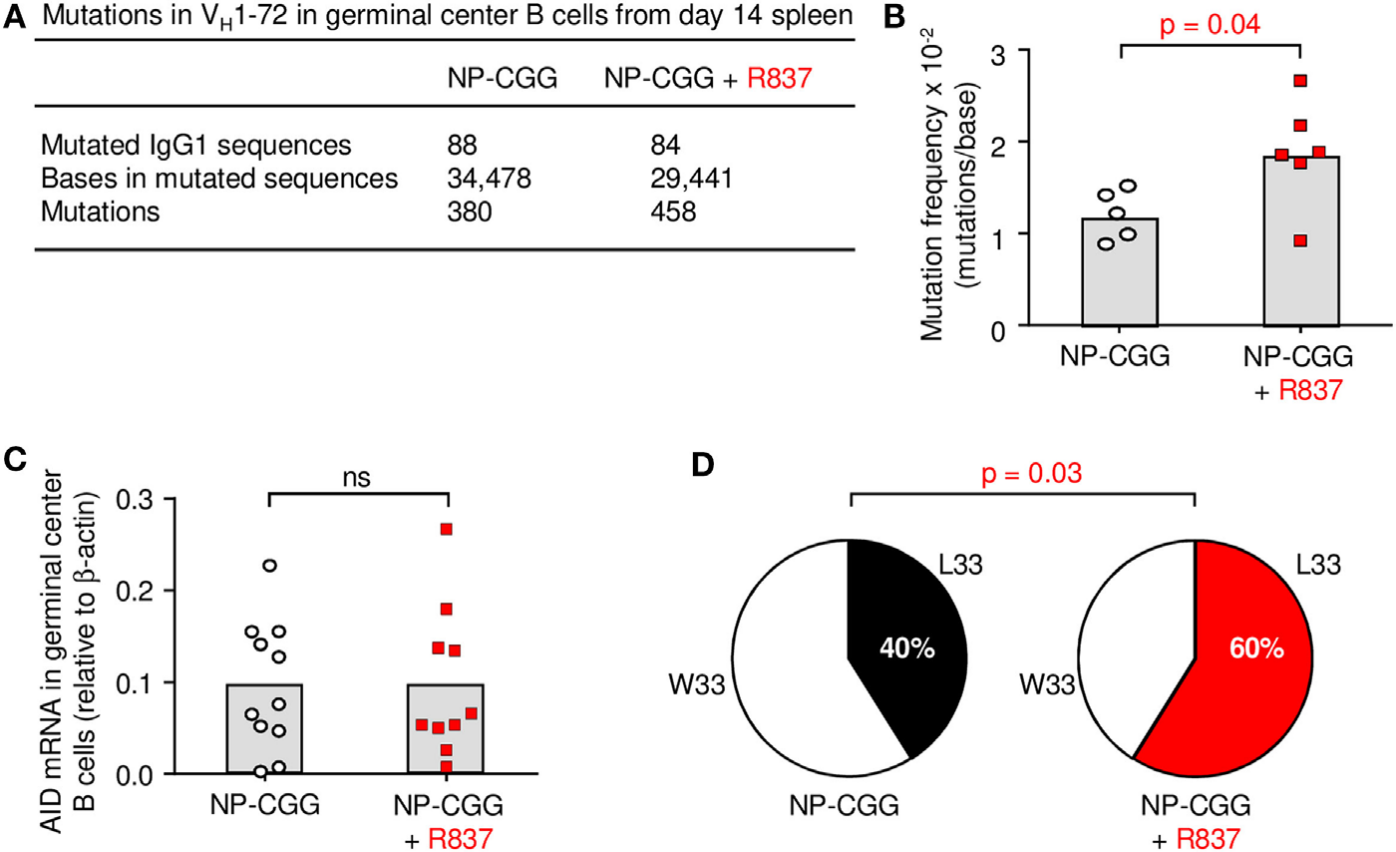
Toll-like receptor 7 enhances somatic hypermutation and affinity in germinal centers by day 14. **(A)** Rearranged V_H_1-72 sequences joined to Cγ1 from germinal center B cells were analyzed for nucleotide mutations. **(B)** Mutation frequencies are depicted; each symbol represents the frequency from one mouse in five to six independent experiments per group. Column indicates mean; *p*-value measured by two-tailed Student’s *t*-test. **(C)** AID mRNA expression. Each symbol represents an individual mouse from five independent experiments with two to three mice each. **(D)** Percent sequences with W33 (low affinity) and L33 (high affinity) mutations are shown in a pie chart. *p*-Value by two-tailed Fisher exact test.

Although the number of germinal center B cells did not change with the inclusion of R837, qualitative differences were found in their BCRs by day 14. Germinal center B cells were isolated by flow cytometry; their purity is periodically assessed and is ~96% pure after sorting (24). V_H_ genes in these isolated cells were then sequenced. The canonical V_H_ gene for binding to NP in C57BL/6 mice is V_H_1-72 using IMGT nomenclature (25), which was formerly referred to as V_H_186.2 (26). Sequencing of the IgG1 receptors revealed a significant increase in somatic hypermutation in the V_H_1-72 exon in B cells receiving signals from antigen and R837 (Figures 3A,B). This was not due to increased AID expression at this time point (Figure 3C), suggesting that the mutation machinery was not altered. Furthermore, the V_H_1-72 gene can undergo a 10-fold increase in affinity with a single amino acid substitution from tryptophan to leucine at position 33 (W33L) in complementarity-determining region 1 (27, 28). Notably, there was a targeted increase in W33L substitutions, indicating selection for high-affinity receptors (Figure 3D).

### Memory Generation Depends on TLR7 Expression in B Cells

**Figure 4:**
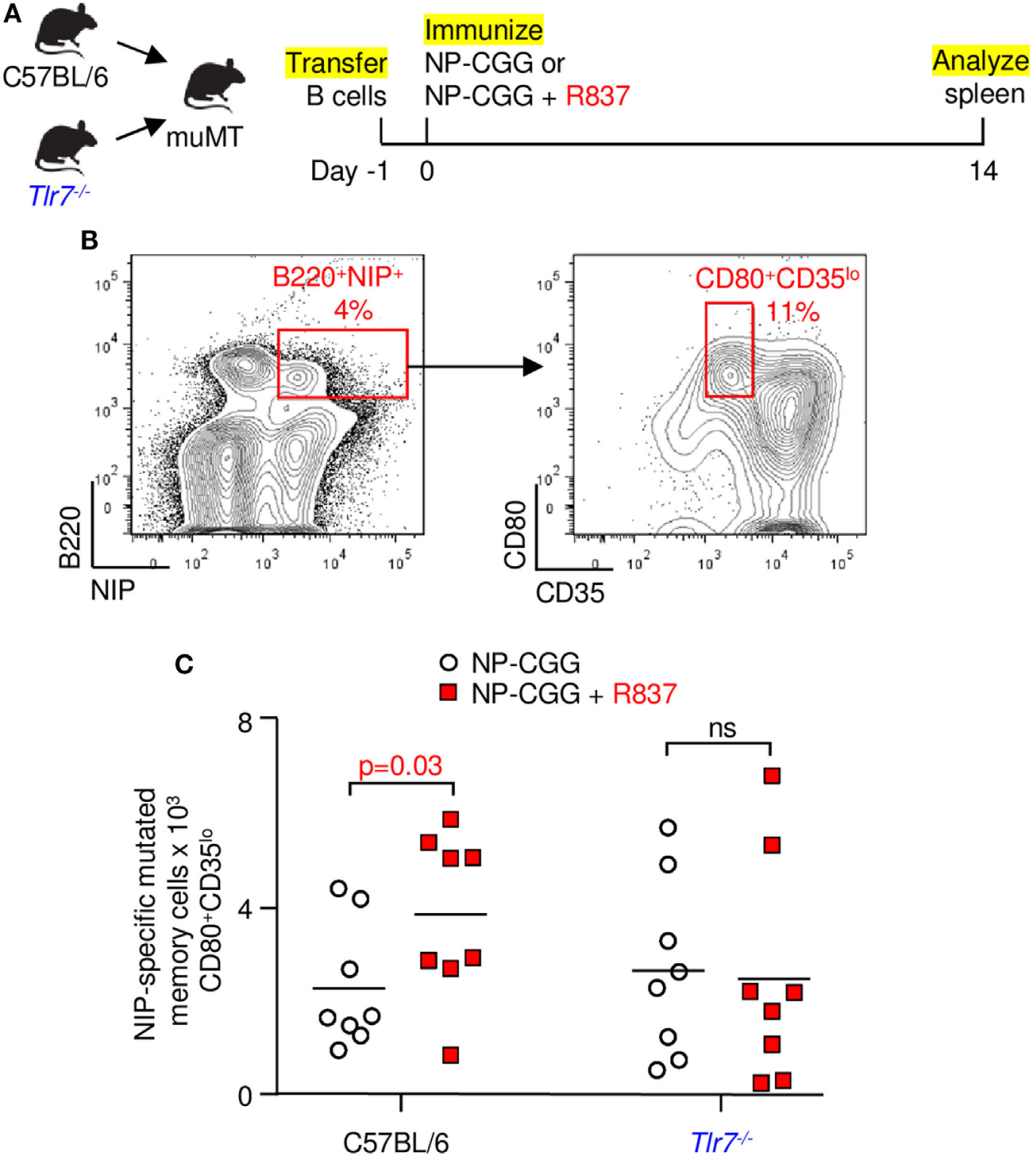
Memory generation depends on toll-like receptor 7 signaling in B cells. **(A)** Splenic B cells from C57BL/6 or *Tlr7^−/−^* mice were transferred into muMT recipients. One day later, mice were immunized, and spleens were analyzed at 14 days. **(B)** NIP-binding B cells were gated for mutated memory cells (B220^+^NIP^+^CD80^+^CD35^lo^). **(C)** Memory B cells from C57BL/6 or *Tlr7^−/−^* donors. Each symbol depicts one muMT mouse from four independent experiments with two mice each. Bar indicates mean; *p*-value determined by one-tailed Student’s *t*-test.

To assess if the mutated B cells were associated with a memory phenotype, naïve B cells from C57BL/6 and *Tlr7^−/−^* mice were transferred into muMT recipients, which do not have mature B cells, and immunized with NP-CGG in the absence or presence of R837 (Figure 4A). After 14 days, splenic B cells were selected with NIP, which binds to antibody better than NP (29), and antigen-specific cells were then stained with CD80 and CD35 (Figure 4B). The CD80^+^CD35^lo^ population has been used to identify mutated memory cells (30). As shown in Figure 4C, signaling through TLR7 in B cells from C57BL/6 donors was required for amplification of mutated memory cells, because the increase in cell numbers was obliterated in *Tlr7^−/−^* B cells. This verified that the enhanced memory response was intrinsic to TLR7 on B cells and not on other immune cells.

### Robust Generation of Antibody Responses after Memory Recall in R837-Immunized Mice

**Figure 5:**
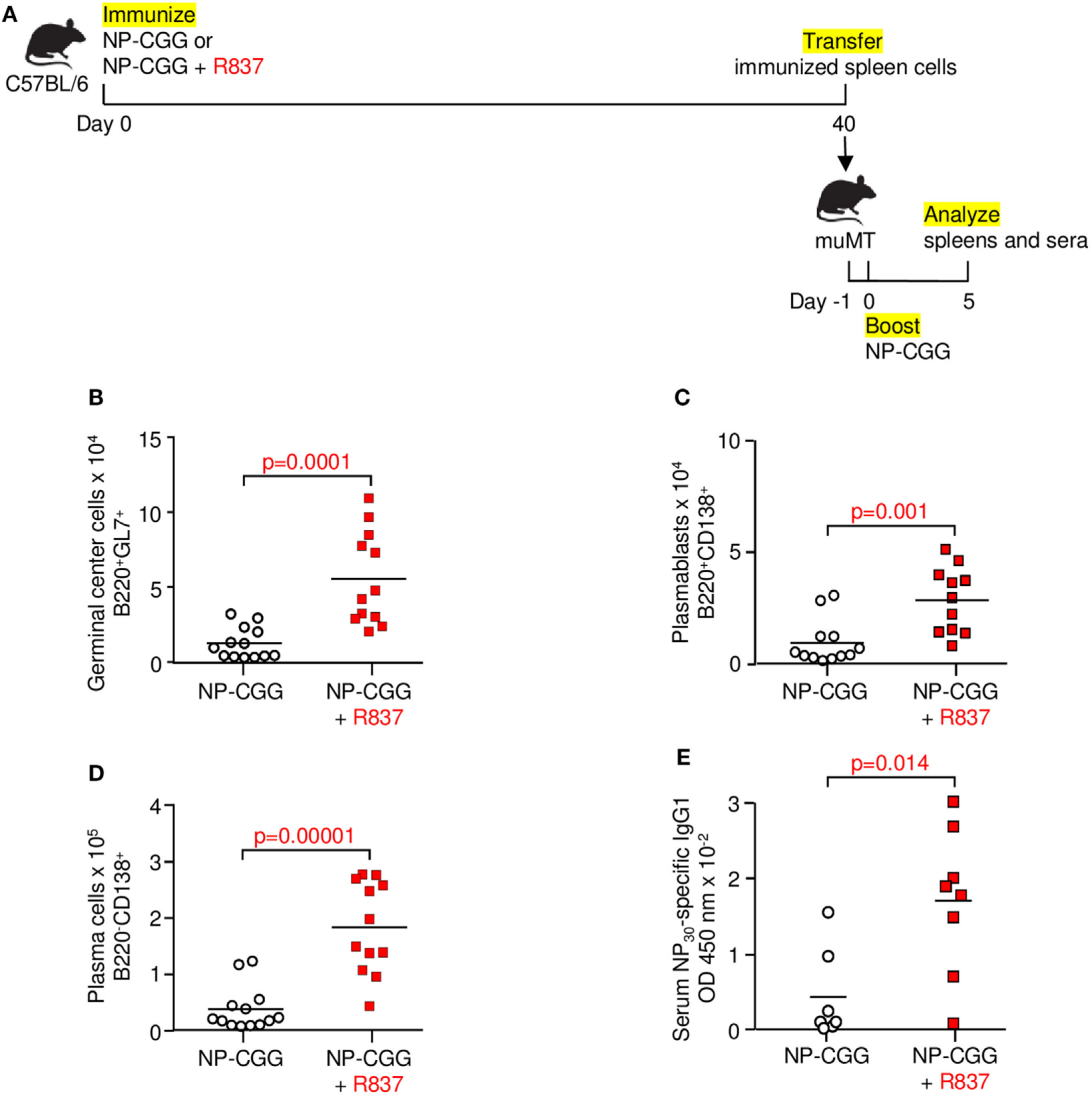
Toll-like receptor 7 signaling augments memory function during recall responses. **(A)** Immunization and adoptive transfer scheme for secondary challenge. Mice were immunized with NP-CGG or NP-CGG plus R837. Forty days later, spleen cells were adoptively transferred into muMT mice. One day after transfer, recipients were boosted with NP-CGG, and spleens and sera were analyzed 5 days later. **(B)** Germinal center cells. **(C)** Plasmablasts. **(D)** Plasma cells. **(E)** Serum NP-specific IgG1 bound to NP_30_-BSA. Each symbol represents a muMT recipient mouse from four independent experiments with one to three mice each. Bar indicates mean; *p*-value determined by two-tailed Student’s *t*-test.

The definition of memory is recalling what has been learned. To determine if the memory cells identified by surface markers and flow cytometry described above actually behave in a recall-dependent fashion, we immunized C57BL/6 mice with NP-CGG in the absence or presence of R837. After 40 days, spleen cells were transferred into muMT recipients. One day later, the recipients were challenged with NP-CGG, and immune responses were measured after 5 days (Figure 5A). When the initial immunization included R837, the secondary response was significantly elevated in terms of increased germinal center B cells (Figure 5B), plasmablasts (Figure 5C), plasma cells (Figure 5D), and total NP-specific IgG1 from serum (Figure 5E). Thus, TLR7 signaling magnified the recall responses by memory B cells, indicating that these were indeed vigorous B cells compared with their counterparts immunized with antigen alone.

## Discussion

To dissect the effect of co-stimulation of B cells through the BCR and TLR7, we systematically examined the primary and secondary responses to a simple hapten-carrier antigen and a synthetic ligand. During the early and late primary responses, co-immunization with NP-CGG and R837 had no measurable enhancement on the frequency of germinal center cells or antibody-secreting cells. Our results differ from other reports that show an augmentation of antibody secretion with TLR7 ligation during the primary response to viruses, which could be attributed to the context of the antigen as single-strand RNA, and may be more intense than stimulation with a synthetic ligand (13–16). Likewise, the viral form of a TLR9 ligand was shown to be critical for optimal B cell responses, compared with soluble CpG ligand with a protein antigen (31). Recent studies have highlighted the efficacy of conjugating CpG to cationic lipids to promote B cell responses through TLR9 signaling (32, 33). Therefore, the physical form of the antigen is crucial. Protein antigens with multiple arrays of a hapten can crosslink the immunoglobulin receptor and negate the necessity for TLR signaling (34), whereas viral antigens with fewer repetitive determinants may rely more on interaction between the BCR and TLR. We propose that simply adding a ligand like R837 as an adjuvant with protein antigens is not sufficient to amplify B cell responses during primary immunizations. A similar conclusion was reached in studies of co-immunization with TLR4 ligands and protein antigens (3, 11, 12).

However, during the secondary response, marked changes were observed. Memory cells generated after antigen priming alone have previously been shown to respond during recall in recipients following adoptive transfer (35–38). Our results reported here reveal a novel role for TLR7 signaling to generate more robust memory B cells, that can be activated after re-encounter with antigen. This is likely a result of favored selection for mutated B cells with higher affinity for NP. We showed that these changes occurred in the niche of germinal centers as early as day 14 in the primary response. The increase in somatic hypermutation and affinity suggests that the mechanism may upregulate MHCII presentation on B cells to vie for T follicular helper cells to drive division, survival, and selection into memory cell precursors (39–41). Memory formation was intrinsic to TLR7 on B cells, and demonstrate that increased memory cells can be induced through B cell stimulation alone. However, the results do not rule out co-engagement of the TLR7 receptor on dendritic cells and macrophages in wild-type mice. Thus, our results indicate that TLR7 signaling regulates selection in germinal center B cells and generates more memory cell precursors during the primary response. Although TLR7 co-ligation in the primary stages did not preferentially produce high-affinity antibodies in sera compared with immunization with antigen alone, the data suggest that the germinal center cells differentiated predominantly into memory cell precursors, rather than plasma cells.

Our immunizations were done using alum as an adjuvant, which contains no TLR ligands, unlike complete Freund’s or monophosphoryl-lipid A adjuvants. Alum is frequently used in human vaccines; likewise R837 is manufactured as imiquimod for human use. Although recombinant protein subunits are rapidly replacing viral immunogens as vaccines, they are less immunogenic (42). It would be advantageous to include a TLR7 ligand such as R837 with these protein antigens to produce the desirable memory population for re-encounter with pathogens.

## Ethics Statement

Animal protocols were reviewed and approved by the Animal Care and Use Committee at the National Institute on Aging, NIH.

## Author Contributions

DC designed and performed experiments, interpreted results, and prepared the manuscript. RM performed experiments, and interpreted results. LK helped with experimental design. PG supervised research and wrote the manuscript.

## Conflict of Interest Statement

The authors declare that the research was conducted in the absence of any commercial or financial relationships that could be construed as a potential conflict of interest.
